# Remarks on Dr. Collins' Letter

**Published:** 1825-05

**Authors:** James Guthrie

**Affiliations:** Surgeon.


ART. VI.—*Remarks on* DR. COLLINS' *Letter.* By JAMES GUTHRIE, Surgeon.

IN the London Medical and Physical Journal for February, Dr. Collins has made some remarks on the case of sloughing of the bladder, related by me in the Edinburgh Medical Journal: and to these it is my duty to reply. In the first place, he has stated that the case could not be one of sloughing of the bladder, but of laceration by an indiscreet use of the forceps; and he grounds this opinion on the circumstance of the patient being unable to retain the urine on the day after delivery. This I consider no certain indication of lacerated bladder. The incontinence of urine after delivery must be attributed to the bladder having lost its natural power of expulsion, both from previous over-distention, and from the injury done to the sphincter and neighbouring parts by the protracted state of the labour.

I repeat, that the instruments had no concern in producing the fistulous opening.

The oval, or almost circular, shape of the aperture, evidently indicating loss of substance, militates so strongly against the idea of laceration, that no intelligent practitioner could possibly mistake it. In fact, the offensive smell which this patient had, both prior to and after delivery, indicated so certainly the existence of sloughing, that I think it perfectly unnecessary to adduce more evidence in support of it. The separation of the slough occurred on the sixth or seventh day after parturition.

2. Dr. Collins next asks, in cases where it is impossible to introduce a catheter, how can any rational practitioner expect successfully to introduce the forceps? To this I reply, that the head of the child may be so jammed betwixt the pubis and sacrum, as to prevent the introduction of the catheter; yet I apprehend that, under such circumstances, little difficulty will be experienced in introducing the forceps; because the head of the child, when it has descended so far as to rest on the perineum, will occupy the conjugate diameter of the pelvis: consequently, sufficient space will be left in the lateral diameter for the successful introduction of the forceps.

Professor Burns, in his Principles of Midwifery, states that, in cases where the catheter cannot be introduced, delivery must be accomplished,—by what?—by instruments, certainly. Now this is at once admitting the impracticability of introducing the catheter, and the practicability of introducing the forceps.

3. The fate of the child, Dr. Collins says, is not mentioned. For information on this point, I refer him to the original case, which it would appear, from this assertion, he has never consulted. I may, in conclusion, inform him, that the woman was delivered of a dead and putrid child, after a labour of three days' continuance.

*Kilmarnock; March 5th,* 1825.

